# Childhood vaccine refusal and what to do about it: a systematic review of the ethical literature

**DOI:** 10.1186/s12910-023-00978-x

**Published:** 2023-11-08

**Authors:** Kerrie Wiley, Maria Christou-Ergos, Chris Degeling, Rosalind McDougall, Penelope Robinson, Katie Attwell, Catherine Helps, Shevaun Drislane, Stacy M Carter

**Affiliations:** 1 Sydney School of Public Health, The University of Sydney, Edward Ford Building A27, Sydney, 2006 Australia; 2 Australian Centre for Health Engagement, Evidence and Values, The University of Wollongong, Wollongong, 2522 Australia; 3 Melbourne School of Population and Global Health, The University of Melbourne, Melbourne, 3010 Australia; 4 School of Social Sciences, Asian Studies & Politics, International Relations, University of Western Australia, Perth, 6009 Australia

**Keywords:** Vaccination, Immunization, Vaccine refusal, Parents, Systematic review, Normative literature, Bioethics, Medical ethics

## Abstract

**Background:**

Parental refusal of routine childhood vaccination remains an ethically contested area. This systematic review sought to explore and characterise the normative arguments made about parental refusal of routine vaccination, with the aim of providing researchers, practitioners, and policymakers with a synthesis of current normative literature.

**Methods:**

Nine databases covering health and ethics research were searched, and 121 publications identified for the period Jan 1998 to Mar 2022. For articles, source journals were categorised according to Australian Standard Field of Research codes, and normative content was analysed using a framework analytical approach.

**Results:**

Most of the articles were published in biomedical journals (34%), bioethics journals (21%), and journals that carry both classifications (20%). Two central questions dominated the literature: (1) Whether vaccine refusal is justifiable (which we labelled ‘refusal arguments’); and (2) Whether strategies for dealing with those who reject vaccines are justifiable (‘response arguments’). Refusal arguments relied on principlism, religious frameworks, the rights and obligations of parents, the rights of children, the medico-legal best interests of the child standard, and the potential to cause harm to others. Response arguments were broadly divided into arguments about policy, arguments about how individual physicians should practice regarding vaccine rejectors, and both legal precedents and ethical arguments for vaccinating children against a parent’s will. Policy arguments considered the normative significance of coercion, non-medical or conscientious objections, and possible reciprocal social efforts to offset vaccine refusal. Individual physician practice arguments covered nudging and coercive practices, patient dismissal, and the ethical and professional obligations of physicians. Most of the legal precedents discussed were from the American setting, with some from the United Kingdom.

**Conclusions:**

This review provides a comprehensive picture of the scope and substance of normative arguments about vaccine refusal and responses to vaccine refusal. It can serve as a platform for future research to extend the current normative literature, better understand the role of cultural context in normative judgements about vaccination, and more comprehensively translate the nuance of ethical arguments into practice and policy.

## Introduction

Vaccine rejection has existed for as long as vaccines [1]. Despite the significant contribution of childhood vaccination to reductions in global child morbidity and mortality [2], some parents continue to reject vaccines for their children. Parents’ reasons for rejection vary widely, and often depend on their social settings. For example, in high-income settings where around 2–3% of parents reject routine childhood vaccines [3, 4], reasons can include previous bad experiences with vaccines or the medical system, concerns about vaccine safety, doubt about the effectiveness or necessity of vaccines, alternative health approaches, and participation in particular social groups or communities. These reasons can be grounded in deeply held religious beliefs or general philosophical approaches to health, views on freedom of choice, or mistrust in government and/or the vested interests of vaccine producers, among other things [5–8].

Vaccination plays a dual role in disease prevention: it serves to protect the vaccinated individual from disease, and when vaccination rates reach a high enough threshold for some diseases, also protects the broader community—including those who remain unvaccinated—by disrupting disease transmission through herd immunity. This dual role of vaccination, providing benefit to both the individual and community, complicates ethical questions regarding vaccine refusal, specifically, whether vaccine rejection is ethically justifiable.

Health care providers, communities, and governments encourage uptake and discourage vaccine rejection by various means, and the dual role of vaccination is also relevant to an evaluation of these practice and policy responses. Vaccine acceptance is encouraged with interventions like incentives, health provider recommendations and “nudges” directed at individual families, as well as by facilitating easier access to vaccination through strategies such as cost reduction and making clinic locations and opening times convenient, with many of these interventions supported by varying levels of evidence [9]. Governments often discourage vaccine rejection via the imposition of mandates that can vary in type and severity [10] and are not always well-supported by evidence [11]. These can include punitive measures, such as limiting unvaccinated children’s access to early childhood education or daycare. A thorough understanding of the ethical dimensions of childhood vaccine rejection and responses to it is important when navigating vaccine rejection in the clinical setting, and when formulating policy [12]. Systematic reviews of the evidence are considered best practice for informing vaccine practice and policy however, to our knowledge there have not yet been any published systematic reviews of the literature on the ethics of childhood vaccine rejection despite there being a broad literature on the subject. We sought to systematically explore and characterise the normative arguments made about parental refusal of routine vaccination, with the aim of better informing vaccine policy and practice.

## Method

**Fig. 1:**
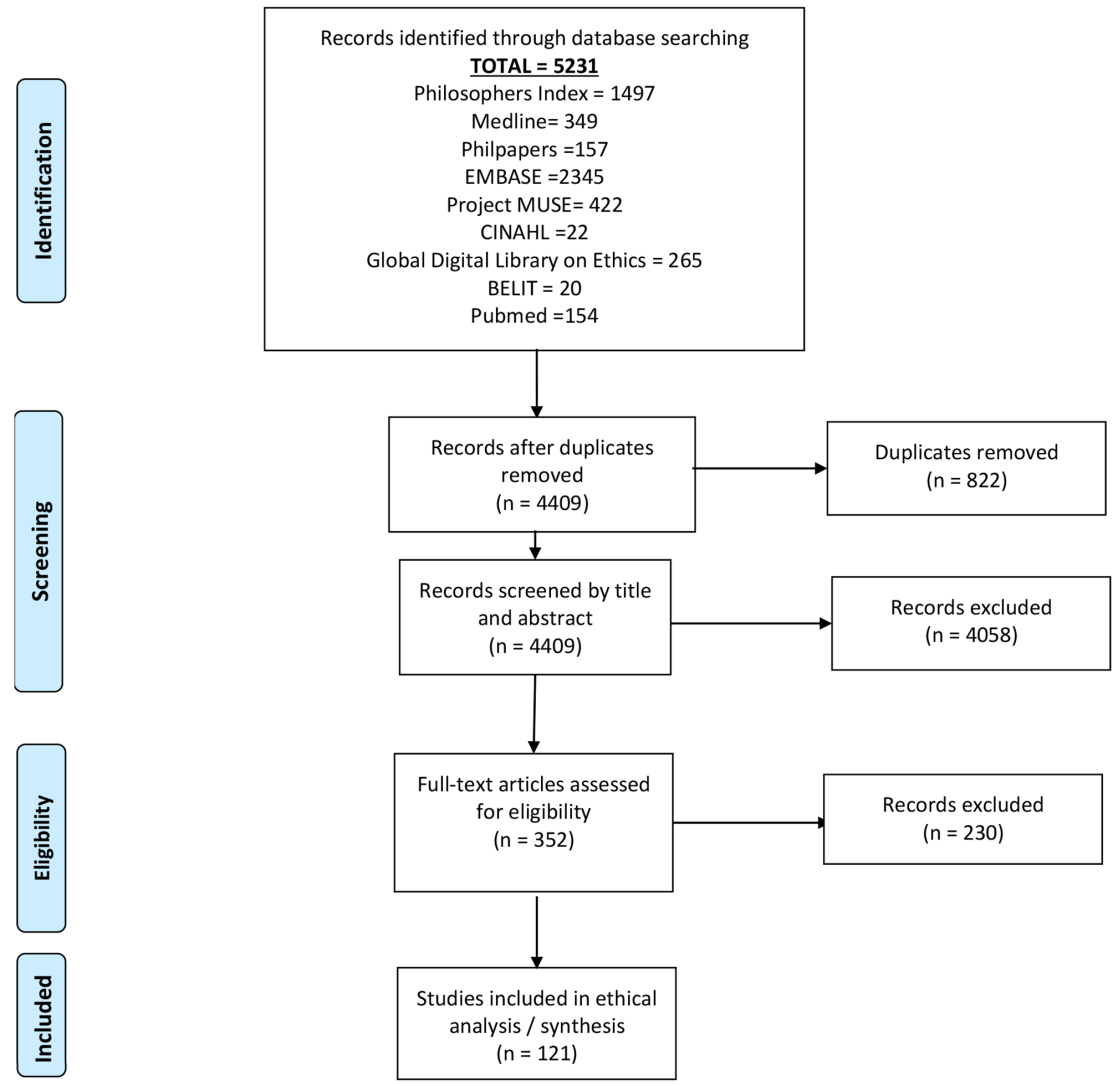
PRIMSA Flow Diagram of Review

We searched nine databases for literature that discussed normative positions on childhood vaccine rejection. Refer to the PRISMA flow chart (Fig. 1.)

### Search strategy

**Fig. 2:**
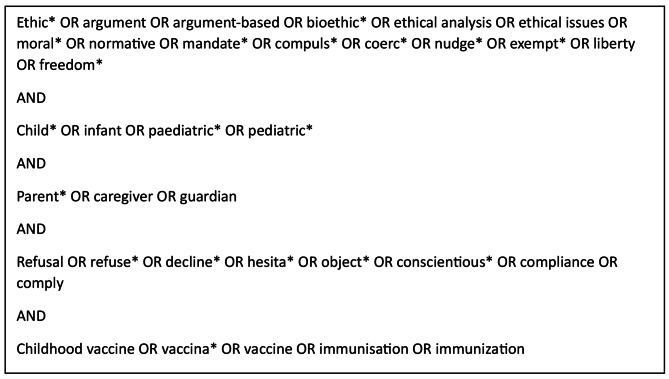
Search strategy

We searched Medline, Embase, Philosophers Index, Philpapers, Project Muse, Cinahl, The Global Digital Library on Ethics (globethics.net), The Bioethics Literature Database (BELIT), and Pubmed using the general search strategy listed in Fig. 2 for articles published between January 1998 and March 2022.

### Inclusion criteria

We included any publication which provided a substantive normative argument about parental refusal of routine vaccines for children aged five and under. We used a broad definition of ‘normative’ to mark anything that goes beyond mere description to consider right and wrong, good and bad, justifiable and unjustifiable, or legitimate and illegitimate actions or ways of being in the world. Our broad conception included textual forms such as ethical reflections, prudential and legal norms, and accounts of rationality. We used ’substantive’ to mark publications where the authors’ main purpose was to make an argument about whether vaccine refusal is morally justifiable. This included empirical research that explicitly examined normative dimensions of vaccine refusal. We were limited to reviewing publications published in English.

### Exclusion criteria

We excluded publications where authors made a normative claim in passing, but the publication’s main purpose was to report non-normative empirical findings. We also excluded: publications on adult vaccination (including COVID vaccination) and the HPV vaccine (which is administered in adolescence, not childhood); empirical research such as surveys or interviews, unless they expressly explored normative arguments; and descriptive publications about the characteristics of the anti-vaccination movement that provided no normative position.

### Screening and data extraction

After search execution and duplicate removal, a screening triangulation exercise was undertaken to ensure consistency among the screeners. A set of 20 titles and abstracts were independently screened by six authors, and the results compared. The inclusion and exclusion criteria were refined in a subsequent group discussion, and a sub-set of full text articles were then screened and evaluated by the same group of people, and results again compared. A discussion of this second triangulation step resulted in a refined and standardized screening approach.

The authorship group were then divided into four pairs, and the remaining titles and abstracts divided among the pairs. Each individual screened titles and abstracts against inclusion criteria, and then met with their screening partner to compare results and discuss and resolve any differences.

Full text was sought for each record screened for inclusion, and a second screening then removed articles which didn’t meet the inclusion criteria once the full text was read, articles that could not be sourced, and duplicates not identified in the initial screening.

The final list of full text publications was then divided among four authors (SC, RM, CD and KW) for data extraction using the concept of “information units” described by Mertz and colleagues [13]. In this context an information unit was defined as a normative issue or argument, and each of the four ‘extracting’ authors summarized each of the relevant information units in the papers they were assigned.

**Fig. 3:**
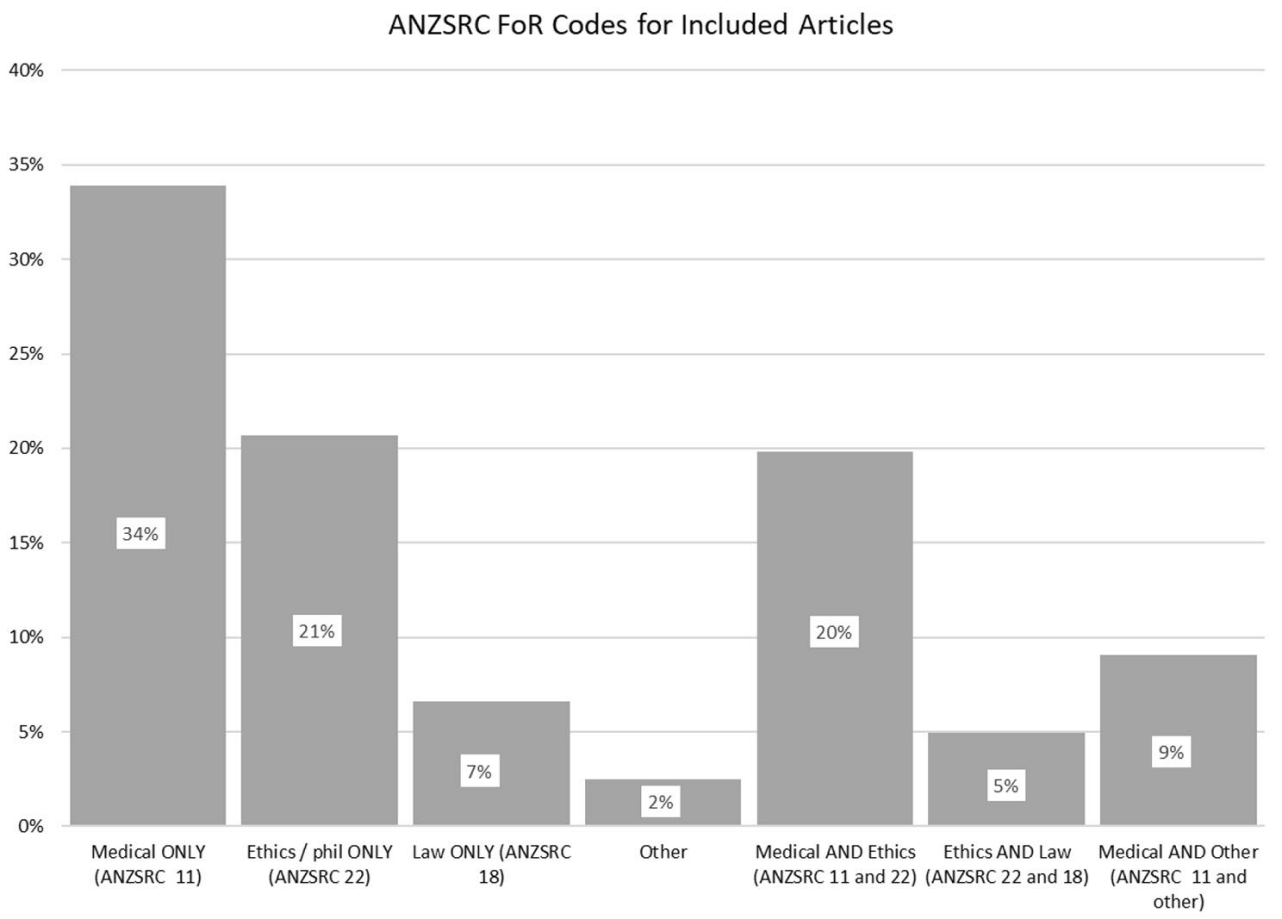
Respective percentages of included articles falling under various ANZSRC FoR Codes (2008)

For included journal articles, Australian Standard Field of Research (FoR) codes for the journal that each article appeared in were sourced as a proxy for the disciplinary location of the article (e.g. bioethics, medicine, law). We used the Australian and New Zealand Standard Research Classification (ANZSRC) 2008, as this was the current standard when analysis commenced [14]. We used two digit FoR codes (division codes) to identify the source journal as either being Medical and Health Sciences (code 11), Ethics and Philosophy (code 22) Law (code 18) or other codes grouped as “other”. In some cases, the journal was assigned a combination of these codes (refer to Fig. 3).

Quality assessment in systematic reviews of normative literature remains a contested area, with various options and no established best practice approach [15]. In this review, we took a satisficing approach to quality appraisal [16]: publication in a peer-reviewed journal or by a reputable academic publisher was taken as a sufficient level of quality to justify inclusion in the review. The peer review process undergone by PhD theses was also taken to be a sufficient indictor of quality to justify inclusion. Further quality appraisal of individual publications was not undertaken. This aligns with the purpose of the review which was to map and synthesize the current literature on this topic.

### Analysis

A framework approach was used to organise and synthesise the data [17]. The extracted information units were read by one author (KW), and a coding frame inductively developed to summarise and classify the information units extracted by the group. The publications were then independently coded according to this framework by two authors (KW and PR). Following this, the two authors met and compared their coding, discussing any differences and resolving them by consensus. The data were then synthesized into themes. In addition, for journal articles, the ANZSRC Field of Research codes for the journal each article appeared in were descriptively analysed to assess the distribution of the included literature across various disciplines.

## Results

### Search results

Five thousand, two hundred and thirty-one publications were returned by the searches (see Fig. 1). Eight hundred and twenty-two duplicates were removed in the first instance, leaving 4409 records to be screened by title and abstract. During this screening process 4058 records were excluded, leaving 351 full text publications to be assessed. Of these a further 230 records were excluded (due to not meeting the inclusion criteria, previously unidentified duplicates, or inability to source the full text), leaving 121 publications for inclusion in the review. These included 117 journal articles, three theses and one book.

### Literature source type

Analysis of the ANZSRC Field of Research codes of the source journals of included articles revealed three main areas, or a combination of them (Fig. 3). Around half were coded to medicine (63%); of these, just over half were dual coded to ethics (20%) or another code (9%). 21% of articles were from the philosophy or ethics literature alone; another 25% were from ethics and medicine or ethics and law. Law was the least dominant discipline, with only 12% of articles being coded to law (alone or in combination with other disciplines). This pattern suggests active concern within medicine regarding non-vaccination, but also widespread overlap in concern between medicine, ethics, and law.

### Main themes found in the literature

Articles addressed two central questions (see Table 1):

1. Whether vaccine refusal was justified (henceforth ‘refusal’ arguments).
2. Whether various policy or practice responses to those who reject vaccines are justified (henceforth ‘response’ arguments).

**Table 1:** Papers included in the review

Reference	Author(s)	Year	Journal	Volume(issue), pages
1	J. Brennan	2018	Journal of Medical Ethics	44(1), 37–43
2	M. J. Walker, S. Clarke and A. Giubilini	2017	Bioethics	31(3), 155–161
3	M. C. Navin	2017	HEC Forum	29(1), 43–57
4	J. Flanigan	2017	Journal of Value Inquiry	51(1), 199–202
5	J. J. Delaney	2017	American Journal of Bioethics	17(4), 56–57
6	E. Parasidis and D. J. Opel	2017	American Journal of Public Health	107(1), 68–71
7	B. J. Christiaan	2017	Journal of Bioethical Inquiry	14(3), 375–384
8	P. J. Carson and A. T. Flood	2017	American Journal of Bioethics	17(4), 36–43
9	M. J. Deem	2017	Nursing	47(12), 11–14
10	D. J. Opel, J. L. Schwartz, S. B. Omer, R. Silverman, J. Duchin, E. Kodish, D. S. Diekema, E. K. Marcuse and W. Orenstein	2017	JAMA Pediatr	171(9), 893–896
11	K. R. W Matthews	2016	Narrative Inquiry in Bioethics	6(3), 172–173
12	T. Kuntz	2016	Narrative Inquiry in Bioethics	6(3), 168–172
13	B. L. Hausman	2016	Narrative Inquiry in Bioethics	6(3), 193–197
14	L. Parker	2016	Narrative Inquiry in Bioethics	6(3), 176–180
15	K. Haller	2016	Narrative Inquiry in Bioethics	6(3), 187–192
16	Josh and J. Mazer	2016	Narrative Inquiry in Bioethics	6(3), 173–176
17	K. Browne	2016	Cambridge Quarterly of Healthcare Ethics	25(3), 472–478
18	T. Ankeney	2016	Narrative Inquiry in Bioethics	6(3), 156–158
19	K. Kirkwood	2016	Narrative Inquiry in Bioethics	6(3), 163–166
20	Committee on practice and ambulatory medicine, committee on infectious diseases, committee on state government affairs, council on school health, section on administration and practice management	2016	Pediatrics	138(3)
21	S. Mann	2016	Journal of the Mississippi State Medical Association	57(7), 216–218
22	J. K. Billington and S. B. Omer	2016	American Journal of Public Health	106(2), 269–270
23	K. S. Hendrix, L. A. Sturm, G. D. Zimet and E. M. Meslin	2016	American Journal of Public Health	106(2), 273–278
24	M. Unterreiner	2016	Journal of Practical Ethics	4(1)
25	R. Griffith	2016	British journal of nursing	25(19), 1076–1077
26	K. Alexander, T. A. Lacy, A. L. Myers and J. D. Lantos	2016	Pediatrics	138(4), 1–6
27	American Academy of Pediatrics	2016	Pediatrics	138(3), 2145
28	K. Stewart	2016	Thesis (Florida Atlantic University)	
29	B. Gray	2016	Clinical Research and Bioethics	7(1), 1,000,256
30	A. L. Caplan and D. R. Curry	2015	Journal of Medical Ethics	41(3), 276–277
31	D. S. Diekema	2015	Journal of Law, Medicine & Ethics	43(3), 654–660
32	J. C. Bester	2015	Journal of Bioethical Inquiry	12(4), 555–559
33	M. J. Smith	2015	Infectious Disease Clinics of North America	29(4), 759–769
34	A. S. Cunningham	2015	BMJ	251, h4576
35	J. M. Glanz, C. R. Kraus and M. F. Daley	2015	Plos Biology	13(8), e1002227
36	L. O. Gostin	2015	JAMA	29(2), 121–130
37	M. Navin	2015	Book: Values and Vaccine Refusal: Hard Questions in Ethics, Epistemology, and Health Care	
38	J. Berlin	2015	Texas medicine	111(9), 22–30
39	R. H. Jeffery	2015	Australian family physician	44(11), 849–852
40	S. L. Block	2015	Journal of Law, Medicine & Ethics	43(3), 648–653
41	H. Y. Lawrence, B. L. Hausman and C. J. Dannenberg	2014	Journal of Medical Humanities	35(2), 111–129
42	T. Dare	2014	HEC Forum	26(1), 43–57
43	J. Flanigan	2014	HEC Forum	26(1), 5–25
44	C. Constable, N. R. Blank and A. L. Caplan	2014	Vaccine	32(16), 1793-7
45	D. J. Opel, K. (A) Feemster, S. (B) Omer, W. A. Orenstein, M. Richter and J. D. Lantos	2014	Pediatrics	133(3), 526 − 30
46	R. Rhodes and I. R. Holzman	2014	Pediatrics	134(Suppl 2), S121-9
47	M. Wicclair	2013	Cambridge Quarterly of Healthcare Ethics	22(3), 308 − 18
48	M. Navin	2013	Public Affairs Quarterly	27(1), 69–85
49	J. L. Schwartz	2013	Human vaccines & Immunotherapeutics	9(12), 2663-5
50	C. A. Rentmeester	2013	Human vaccines & Immunotherapeutics	9(8), 1812-4
51	R. Grifith	2013	Br J Community Nurs	18(11), 545-7
52	D. S. Diekema	2013	Hum Vaccin Immunotherapeutics	9(12), 2661-2
53	J. Blignaut	2013	Thesis: University of Cape Town	
54	D. Ropeik	2013	Human Vaccines & Immunotherapeutics	9(8), 1815–1818
55	A. Caplan	2013	Human Vaccines & Immunotherapeutics	9(12), 2666-7
56	Anonymous author	2012	Medical Ethics Advisor	9
57	K. Insel	2012	The Virtual Mentor	14(1), 17–22
58	A. L. Caplan, D. Hoke, N. J. Diamond and V. Karshenboyem	2012	Journal of Law, Medicine & Ethics	40(3), 606 − 11
59	T. Newman	2012	Minnesota Medicine	95(8), 24–25
60	D. Isaacs	2012	New South Wales Public Health Bulletin	23(5), 111–115
61	J. D. Lantos, M. A. Jackson and C. J. Harrison	2012	Journal of Health Politics, Policy & Law	37(1), 131–140
62	D. J. Opel and D. S. Diekema	2012	Journal of Health Politics, Policy & Law	37(1), 141-7
63	D. Nulty	2011	JONA’s Healthcare Law, Ethics, & Regulation	13(4), 122-4
64	J. L. Schwartz and A. L. Caplan	2011	Primary Care; Clinics in Office Practice	38(4), 717 − 28
65	J. Gilmour, C. Harrison, L. Asadi, M. H. Cohen and S. Vohra	2011	Pediatrics	128(4), S167-74
66	M. Poreda	2011	Seton Hall Law Review	41(2), 765–811
67	D. Isaacs, H. A. Kilham, S. Alexander, N. Wood, A. Buckmaster and J. Royle	2011	Vaccine	29(37), 6159-62
68	A. Finn and J. Savulescu	2011	Lancet	378(9790) 465-8
69	D. S. Diekema	2011	J Clin Ethics	22(2), 128 − 33
70	A. Chatterjee and C. O’Keefe	2010	Expert Review of Vaccines	9(5), 497–502
71	J. D. Lantos, M. A. Jackson, D. J. Opel, E. K. Marcuse, (A) L. Myers and (B) L. Connelly	2010	Current Problems in Pediatric & Adolescent Health Care	40(3), 38–58
72	S. Kling	2009	Current Allergy and Clinical Immunology	22(4), 178–180
73	D. Khalili and A. Caplan	2007	The Journal of Law, Medicine & Ethics	35(3), 471-7
74	B. Halperin, R. Melnychuk, J. Downie and N. Macdonald	2007	Paediatrics & Child Health	12(10), 843-5
75	A. Lyren and E. Leonard	2006	Clin Pediatr (Phila)	45(5), 399–404
76	J. D. Blum and N. Talib	2006	Medicine and Law	25(2), 273 − 81
77	M. Wharton, R. Hogan, P. Segal-Freeman and A. Hinman	2005	The Journal of Law, Medicine & Ethics	33(4), 34–37
78	A. Dawson	2005	Bioethics	19(1), 72–89
79	D. S. Diekema and the Committee on Bioethics	2005	Pediatrics	115(5), 1428-31
80	J. Wood-Harper	2005	Nursing Ethics	12(1), 43–58
81	E. J. Furton	2005	Ethics and medics	30(12), 1–2
82	T. May and R. D. Silverman	2005	Human Vaccines	1(1), 12–15
83	S. P. Calandrillo	2004	University of Michigan Journal of Law Reform	37(2), 353–440
84	E. J. Furton	2004	The National Catholic Bioethics Quarterly	4(1), 53–62
85	H. Baker	2004	Camb Law J	63(1), 49–52
86	P. N. Goldwater, A. J. Braunack-Mayer, R. G. Power, P. H. Henning, M. S. Gold, T. G. Donald, J. N. Jureidini and C. F. Finlay	2003	Medical Journal of Australia	178(4), 175-7
87	P. McIntyre, A. Williams and J. Leask	2003	Medical Journal of Australia	178(4), 150–151
88	A. R. Hinman, W. A. Orenstein, D. E. Williamson and D. Darrington	2002	Journal of Law, Medicine & Ethics	30(3), 122-7
89	J. Froome and K. Badcock	2002	Nursing Times	98(12), 16
90	D. A. Salmon and A. W. Siegel	2001	Public Health Reports	116(4), 289 − 95
91	R. D. Silverman and T. May	2001	Margins	1(2), 505 − 21
92	R. Swan	2000	The Humanist	60(6), 11
93	S. Pywell	2000	Medical Law International	4(3), 223 − 43
94	P. Bradley	1999	Journal of Medical Ethics	25(4), 330-4
95	T. Dare	1998	Bioethics	12(2), 125–149
96	A. Rogers and D. Pilgrim	1995	Health Care Analysis	3(2), 99–107
97	N. J. Ngcobo	2009	Thesis	
98	A. Fernbach	2011	Journal of the American Academy of Nurse Practitioners	23(7), 336 − 45
99	Deem, M. J., Navin, M. C., & Lantos, J. D.	2018	JAMA Pediatrics	172(6), 514–516
100	Rossi, R., Rellosa, N., Miller, R., Schultz, C. L., Miller, J. M., Berman, L., & Miller, E. G.	2020	Pediatrics,	146(4), e20200768
101	Bester, J. C.	2018	Clinical pediatrics	57(5), 505–508
102	Hadjipanayis, A., Dornbusch, H. J., Grossman, Z., Theophilou, L., & Brierley, J.	2020	European Journal of Pediatrics	179, 683–687
103	Kennedy, J.	2020	Perspectives in public health	140(1), 23–24
104	Kling, S	2020	Current Allergy and Clinical Immunology,	33(1), 48–51
105	Aorora, K. S., Morris, J., & Jacobs, A. J.	2018	Journal of Clinical Ethics	29(3), 206–216
106	Giubilini, A., Caviola, L., Maslen, H., Douglas, T., Nussberger, A. M., Faber, N.,. . Savulescu, J.	2019	HEC Forum	31(4), 325–344
107	Zagaja, A., Patryn, R., Pawlikowski, J., & Sak, J.	2018	Medical Science Monitor	24, 8506–8509
108	Blunden, C. T.	2019	BMJ	45, 71–74
109	Bock, G. L.	2020	Journal of Medical Ethics	46, 114–117.
110	Horan, R. A.	2019	Awards for Excellence in Student Research and Creative Activity – Documents. 7	
111	Pierik, R.	2018	Journal of applied philosophy	35(2), 381–398
112	Navin, M. C., & Attwell, K.	2019	Bioethics	33(9), 1042-49
113	Haire, B., Komesaroff, P., Leontini, R., & MacIntyre, C. R.	2018	Bioethical Inquiry	15(2), 199–209
114	Williamson, L., & Glaab, H	2018	BMC Medical Ethics	19, 84
115	Giubilini, A., Douglas, T., & Savulescu, J.	2018	Medicine, Health Care and Philosophy	21(4), 547–560
116	MacDonald, N. E., Harmon, S., Dube, E., Taylor, B., Steenbeek, A., Crowcroft, N., & Graham, J.	2018	Paediatr Child Health,	24(2), 92–97
117	Weithorn, L. A., & Reiss, D. R.	2018	Human vaccines and immunotherapeutics	14(7), 1610-17
118	Tomsick, E.	2020	Journal of Law and Health	34(1), 129–154
119	Rus, M., & Groselj, U.	2021	Vaccines	9(2), 113
120	Bernstein, J.	2021	Kennedy Institute of Ethics Journal	31(1), 17–52
121	O’Neil, J.	2020	Journal of medical ethics	46, 108–111

### Descriptive analysis of content

**Fig. 4:**
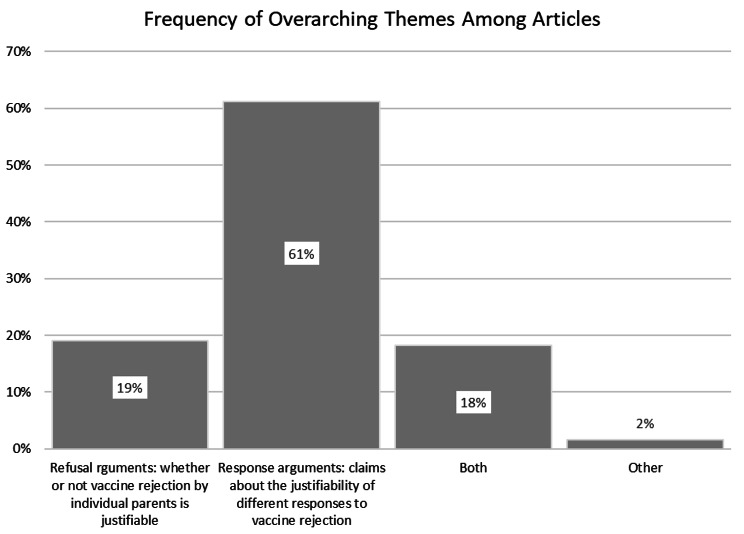
Comparative frequencies of themes occurring among included articles

The literature was dominated by papers focused on ‘response’ arguments (61%). A smaller group of papers address ‘refusal’ arguments (19%), and about 18% considered both ‘refusal’ and ‘response’, usually making normative arguments about vaccine refusal as background to arguments regarding ‘response’ (See Fig. 4). Less than 2% of papers had a different focus.

**Fig. 5:**
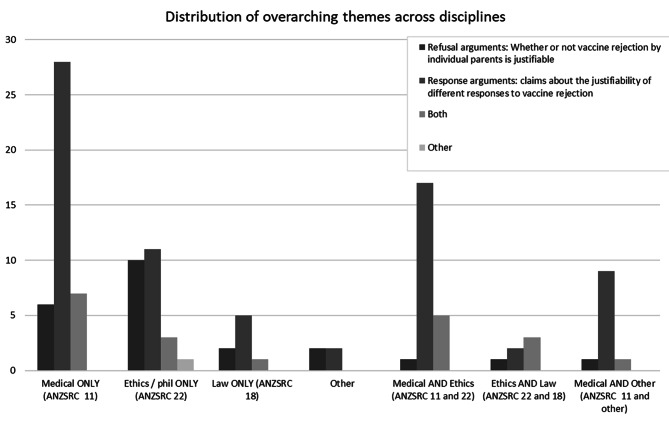
Comparative frequency of overarching themes across the different disciplines of the included articles

‘Response’ arguments were more common in the medical and health sciences literature (ERA FoR code 11, see Fig. 5). Although the ethics/philosophy (FoR code 22) and law literatures (FoR code 18) were also dominated by ‘response’ arguments, these journals—unlike medical journals—were more likely to include ‘refusal’ arguments.

As would be expected, authors made ‘response’ and ‘refusal’ arguments in different ways. In the following sections we consider the detail of how arguments were made. We refer to each included article by its unique reference listed in Table 1.

## ‘Refusal’ arguments: whether or not vaccine rejection by individual parents is justifiable

Arguments about whether vaccine refusal by individual parents is justifiable included consideration of parents’ rights, the interests of the child (including the legal ‘best interests of the child standard’), the value of herd immunity, the epistemic basis for ethical claims, and the relevance of religious views. Our sampling period included a special issue of *Narrative Inquiry in Bioethics* which published narratives written by parents to communicate their normative positions on vaccination. Most of these were written by non-vaccinating parents, and they make up over one third of all arguments in the identified literature that support refusal. On balance, most of the literature argues that it is not justifiable for parents to refuse routine vaccination for their children.

**Fig. 6:**
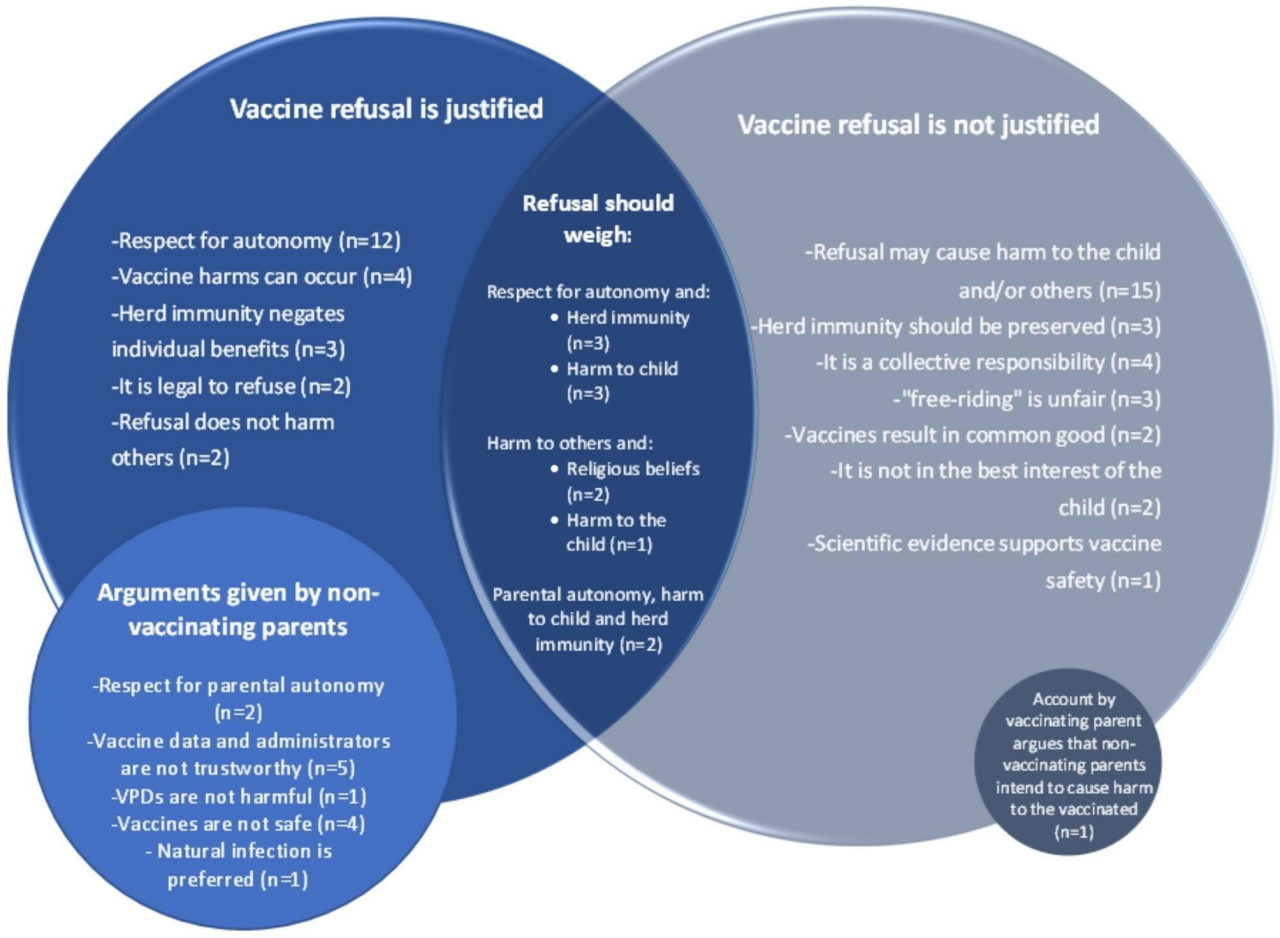
‘Refusal’ arguments made in the literature on the ethics of vaccine refusal

Some arguments within the literature were absolute in their position on whether vaccine rejection is justifiable; others weighed competing values in a situation-specific approach. Irrespective of the arguments used to justify a position, most of the literature frames the question of whether vaccine rejection is justifiable based on three key areas of concern: (i) Respect for autonomy, the doctrine of informed consent and the value of liberty, (ii) Consequences for the child and others, and/or (iii) The normative significance of parental trust, distrust, and uncertainty. We explore the main arguments within these concepts below. As the discussion shows, these concepts are not discrete – they are often weighed against one another, linked by causal claims, or held in tension in the arguments made. Figure 6 represents proportionally the ’refusal’ arguments made in the reviewed literature.

### Respect for autonomy, the doctrine of informed consent and the value of liberty

Fifteen papers from this sample present arguments that vaccine refusal is justified based on respect for parental autonomy, rights, or liberties (21, 23, 25, 31, 32, 35, 36, 39, 68, 71, 75, 80, 94, 100, 121). Some argue that vaccine refusal is justified on the basis of preserving legal rights (31, 80) or expression of religious freedom [23]. Opposing positions (including from four of the authors who also offer arguments justifying refusal) argue that, on balance, considerations regarding respect for autonomy are, or can be, outweighed by the potential harm caused to the child and others by not vaccinating though the increased risk of vaccine preventable diseases (21, 36, 20, 23, 110). This includes legal perspectives arguing that the freedom to choose is not unfettered [25] and that courts can override parental autonomy if this is in the child’s best interest (75, 85), as well as arguments from religious perspectives that the freedom to exercise religious beliefs needs to be weighed against harm caused to others (21,91). Those who argue that vaccine refusal is justified counter that disrespecting parental autonomy can also cause harm to the child through loss of trust and possible disengagement of the child from the healthcare system (100), and that the increased risk of disease is a price worth paying to ensure that political values are preserved (71). Of note: non-vaccinating parents also assert a right to make choices for their children in support of their refusal [14, 18], but unlike others, their arguments are based primarily on epistemic claims about vaccine effectiveness, necessity and safety rather than moral or ethical positions. However, they assert that these doubts necessitate respect for their decision.

### Consequences for others and the child

Most of the literature argues for or against the justifiability of vaccine refusal based on consequences. These include potential harms from vaccine preventable diseases or vaccines themselves, or conversely, potential benefits from herd immunity. The concept of herd immunity is deployed in different ways. Those justifying vaccine refusal in certain circumstances argue that in settings where there is a high level of herd immunity, the risk posed by an unvaccinated child is not great enough to override respect for parental autonomy (62, 65, 94, 98), and that the benefits of community protection do not justify the individual risk posed by the vaccine and borne by the child who is already protected through herd immunity (72, 96, 97, 17, 93, 108). Perspectives of non-vaccinators echo these ideas by asserting that some diseases are not harmful enough to proscribe vaccine refusal [14] and that vaccine injury contributes to and justifies refusal [16].

In contrast, those who argue that refusal is not justifiable propose a duty to contribute to herd immunity because it is a public good (7,80, 19,120, 33, 48, 68,115), or that free-riding (allowing one’s child to enjoy the benefits of herd immunity provided by others, while avoiding the risk of vaccinating) is unfair (37,46, 48). On this account, the vaccine refusal of a few may undermine herd immunity and thus cause harm to the many by increasing disease risks (9, 11, 26, 37, 59, 76, 81, 86); further, these risks are borne by the most vulnerable (43). These arguments about harm to others include those made by authors writing from religious perspectives (8, 81, 84, 92, 98). Finally, an account by a vaccinating parent suggests that harms resulting from non-vaccination are blameworthy because they are an intentional act of aggression against vaccinated children [19].

The concept of the child’s interests arises frequently in these publications. Pursuing or protecting these interests generally combines concern about the consequences of non-vaccination for the child with concern for autonomy, in the broad sense of being able to direct one’s life in accordance with one’s values or aims. Authors write about the interests of the child in both a general sense (i.e. the interests of the child outside of a legal context) and in a legal sense (the formal ‘best interests of the child standard’). The legal construction is used both to support (31, 6, 93) and to oppose vaccine refusal. Arguments that receiving a vaccine is in the legal ‘best interests of the child’ (21,39) posit that any deviation from a widely accepted legal view of the interests of a child should weigh the risk of harm to the child (68) irrespective of the parent’s beliefs (78), or that non-vaccination constitutes negligence or child endangerment [28]. On the other hand, some authors argue that, from a legal perspective, parents have the right to consent to or refuse vaccination ostensibly using the ‘child’s best interests standard’(93) and that there is insufficient legal precedent to argue that non-vaccination constitutes medical neglect [6].

### Arguing from distrust and uncertainty

As previously noted, the sample included a set of papers written from the perspective of non-vaccinating parents. Most of these contributions seek to justify vaccine refusal, and many justifications were grounded in distrust. They call into question vaccine safety and effectiveness [12–14, 18], and the accuracy of the reporting of adverse events following immunization (96). They claim financial conflicts, constructing clinicians, clinical medicine, and/or regulatory agencies as untrustworthy or non-credible [12, 14, 16]. They cite empirical studies of non-vaccinators to support parental preferences for natural infection over a vaccine (97). Non-vaccinating parents were not the only authors to make arguments in this vein. Some other authors cite the lack of absolute certainty of vaccine safety as justification for parents refusing vaccines in the interests of their children (28,76), especially regarding newer vaccines for which efficacy is not well-established (34). This line of argument depicts vaccine proponents as driven by commercial interests, thus justifying parental mistrust and refusal (34). Contra this, one paper asserts that refusal on the grounds of mistrust of government or medicine is not justifiable, as it is inconsistent with the scientific evidence and the well-established regulatory processes in place, such as the rigorous clinical testing required to develop and approve vaccines, and the systems established to report adverse events and ensure safety [8].

## ‘Response’ arguments: claims regarding the justifiability of different responses to non-vaccination

**Fig. 7:**
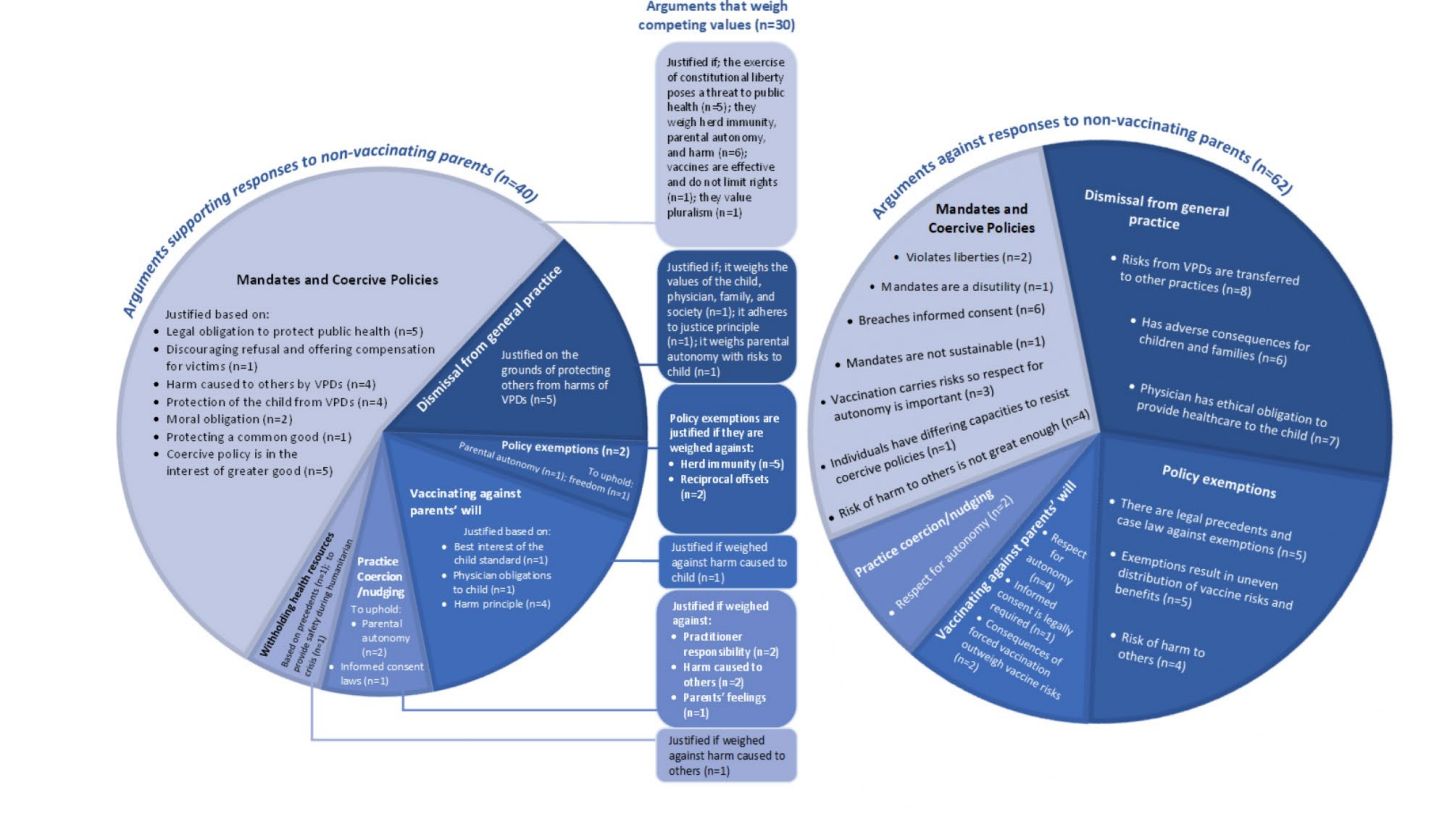
‘Response’ arguments made in the literature on the ethics of vaccine refusal

The literature examines four main responses to non-vaccination (i) government mandate policies (such as legal ramifications for refusing vaccination and vaccination as a school entry requirement), and other coercive policies, (ii) exemptions to mandate policies, (iii) individual practitioner and medical practice responses (including patient dismissal from practice for vaccine refusal, vaccinating against parents’ will, and nudging), and (iv) withholding health resources. The literature includes authors who argue that these responses are justifiable and others who argue that they are not. Much like the refusal arguments, some response arguments are absolute in their position, while others advocate weighing competing values in a context -specific way. Like refusal arguments, most arguments for and against particular responses to non-vaccinating parents draw from respect for autonomy, the doctrine of informed consent and the value of liberty, as well as considering consequences for the child and others. Other concepts appearing in these arguments include inequity, and the duties of governments and practitioners. Figure 7 represents proportionally the ’response’ arguments made in the reviewed literature.

### Respect for autonomy, the doctrine of informed consent and the value of liberty

As in the literature on refusal, many arguments about policy or practice responses to non-vaccinating parents depend on the interrelated concepts of respect for autonomy, informed consent and liberty. Five papers engage with the issue of practitioners vaccinating against parents’ will with respect to these concepts. They argue that forced vaccination by healthcare providers violates parents’ autonomy and/or the ethical requirement for informed consent, because vaccination carries risks (80,119), and clinicians have legal obligations to obtain valid consent for procedures (94). Some authors propose alternatives to forced vaccination, including focusing on rebuilding trust (rather than violating negative liberty) (32), and accepting that views on vaccination derive from plural and culturally-specific values [29]. On the other hand, proponents of forced vaccination do not engage with these concepts, instead deploying the harm principle and the legal ‘best interests of the child standard’ to justify their position. We explore this argument in the following section “Consequences for the child and others”.

Another set of papers make arguments about vaccine mandates that also draw on autonomy or liberty justifications, often weighing these against harm or risk of harm. Arguments justifying mandates are often legal in nature and use, for example, the harm principle or case law to argue that the freedom or liberty to choose not to vaccinate is limited by the risk of ill health and/or death to the child or others in the community, including vulnerable persons (83,91). One author argues that legal actions should be brought against those who harm others by refusing vaccination, as this would both discourage refusal and, in the case of any successful claims, compensate victims (55). Some authors argue that mandates are justifiable if the exercise of liberty rights poses a threat to public health (53,82,83,91,119). While those arguing that mandates are not justifiable sometimes rely on arguments about risk of harm—i.e. that in a low-incidence (and therefore low-risk) setting mandates cannot be justified (45, 87,104)—most make their arguments from autonomy, informed consent, and personal liberty and do not weigh these against the potential for harm (12,16,61,82,89,107,114). One author argues that even if mandates improve vaccination rates, they damage trust with parents and make refusers more steadfast in their decision (121), so are not sustainable. Finally, some authors present middle-ground positions that—in their view—are more autonomy- or liberty-preserving, including persuasion (121) or weakly enforced mandates (71), or argue that policy responses should take the least coercive approach that is feasible and effective to balance the needs of the individual with public health (117).

Those supporting conscientious objection to mandates argue that such provisions contribute to the collective good of a culture of respect for autonomy (82), or reflect the “American ideal” of personal freedom (66). Contra this, those opposed to conscientious objection provisions argue that challenges to mandates based in religious freedom have failed in case law, as the right to practice religion freely does not include the liberty to expose children or communities to disease (20,92). One author provides a qualified view of conscientious objection on religious grounds, arguing that such liberties could be justified only while high vaccination rates are maintained (109).

Authors disagree about whether certain policy or practice responses do, or do not, respect autonomy or uphold important liberties. For example, authors disagree on the effect of both nudges and conscientious objection policies on parental autonomy or liberty. With respect to nudges, some argue they are autonomy-preserving because they steer parents in a certain direction while allowing choice (106), do not override or challenge the strong views of deeply opposed opponents (42, 44) and uphold informed consent (121). Some supporters of nudging weigh multiple normative considerations, arguing that nudges that appeal to social responsibilities in a medical practice setting are justified because they appropriately balance parental autonomy against the practitioner’s responsibility to promote trust and collective benefits (3,80). Those opposed to nudges for vaccination decisions argue that the invasive nature of immunization increases the need for independent and informed decision making (60,113). These authors argue against a presumptive consultation style in general practice, proposing participatory clinical encounters (114), and using persuasion (42), as alternatives to more coercive approaches.

### Consequences for the child and others

Many of the arguments in this literature consider individual and collective consequences—benefits, harms, burdens, and costs to society — and propose that these may override other normative considerations. The risk and prevention of harm is particularly pertinent here. For example, a parental decision can be overruled in cases where there is a significant risk of harm to the child (78), or nudges become more justifiable when the risk of harm to others is higher (3, 75).

Arguments about mandates often include concern about consequences, since it is inherent in a vaccine mandate that there will be some costs associated with non-vaccination. Mandate proponents argue that mandates ensure high vaccination rates, thus preventing disease outbreaks (39) and associated harms (97), so are in the best interest of individual children (28, 73, 111) and serve the greater good (4,28,73,79). Some justify mandates by proposing a duty to contribute to herd immunity, including under the “clean hands principle”, that is, an obligation not to participate in collectively harmful activities [1, 5]. Conversely, some authors argue that mandates are not necessary to achieve high levels of population immunity, so state coercion is unjustified at a collective level or at the level of the individual child because each child receives limited benefit (94). Those opposing mandates also argue that vaccine safety is not absolute (88) and that mandates are a disutility, carrying associated costs with surveillance and enforcement (95). Other authors sought to balance these kinds of consequences against other normative considerations with respect to mandates, including the level of herd immunity, the risks of non-vaccination to the child and/or society, and respect for parental autonomy (32,53,88,119). One author argues that mandates protect ‘victims’ of the anti-vaccination movement from harms so long as certain conditions are met (43): that the vaccine can prevent infection and transmission, that individuals minimize their risk of exposure, and that the right of self-defense is preserved (e.g. in the case of allergy to vaccines).

Consequences are also important to arguments about conscientious objection, but here it is generally concerns about the impact on the collective. Some argue that exemptions should not be allowed because they may increase rates of disease or undermine individual or community health (20, 87, 118); others argue that if disease risk is low, exemptions are justified because those few individuals with exemptions do not pose a risk to others or herd immunity (20, 82, 105).

Consequences to the child and others are used to justify whether responses should be applied in general practice settings. As mentioned in the previous section, some authors justify healthcare workers vaccinating against a parent’s will using both the harm principle (69) and the legal ‘best interests of the child standard’ [25]; others suggest it is against the legal best interests of an older child to be forcibly vaccinated, as this may have a more detrimental impact than being unvaccinated (25,51). The best interests of the child are also invoked extensively to argue that non-vaccinating families should not be dismissed from medical practices (98,104, 26, 75). Here authors note that an unvaccinated child is more vulnerable to vaccine preventable diseases (9, 49), practice dismissal limits opportunities to access health care (31,52, 56,79,116) and the increased risk of harm from vaccine preventable diseases is transferred to other practices (9,47,49). One paper makes an argument about the consequences of treating non-vaccinating families for general practitioners, suggesting that practices caring for unvaccinated children should disclose this to other patients to minimize medicolegal risks, and should receive legal protection to account for the increased liability and risk of caring for these patients (40).

A small body of literature employs claims about who is responsible for the consequences of non-vaccination to make arguments about responses to non-vaccination. For example, one article seeks to justify discriminating against unvaccinated children with a vaccine preventable disease by limiting their access to health resources, relying on precedents such as coronary bypass surgery being withheld from obese people and smokers, and arguing that those who contribute to their own ill-health (in this case by not vaccinating) do not deserve healthcare (80). A related argument focuses on managing refugee camps during outbreaks that pose a direct and imminent threat of harm, proposing that the state is justified in withholding humanitarian aid from non-vaccinating refugees because the state is responsible for setting conditions that provide protection to (or prevent harm to) aid givers and public health [30].

### Inequity

Some critiques of policy or practice responses to non-vaccination emphasise that these responses can have inequitable effects and argue that this is unjustifiable. Exemption policies are a key focus here. Five papers argue against exemptions to vaccine mandates on the grounds that these unevenly distribute the risks and benefits of vaccinations (27,61,66, 73,118). These authors propose that the inaction of a few compromises the health of the most vulnerable community members (118) and disenfranchises those with medical contraindications for vaccines [27]. One author particularly focuses on home-schooled children, arguing that exempting them from vaccine mandates exposes both those children and society to harm, and that it is in the interests of these children and society that they be protected through vaccination (73). Some authors suggest that policy exemptions could be made justifiable by imposing conditions that offset potential inequities. On this view, exemptions could be justified so long as the refuser is prepared to make a financial or other contribution to help offset the potential financial burden of the diseases they may cause, or to otherwise contribute to social good [2, 22].

Similarly, some opponents of coercive mandates or practice dismissal for non-vaccination critique these responses for having inequitable effects. It is argued that coercion risks creating a group of disenfranchised people (113) and that different people have different capacities to resist coercive policies (114). Similarly, dismissal leaves vulnerable children without advocacy (64), leads to patients not being treated equally (63) and marginalizes children from health care (74). One paper argues that family dismissal should be strongly discouraged, and an alternative mutually beneficial solution sought after considering the interests of the patient, physician, family, community, and society at large (74).

### The duty of practitioners and the state

Some papers address the duties of practitioners and the duties of the state to respond to non-vaccination, in ways that go beyond simply weighing up consequences, implications for autonomy or freedom, or equity of impacts.

A variety of duties of practitioners are proposed. The first of these is to protect a child from their parent’s beliefs if those beliefs are likely to cause significant harm, which is used to justify initiating child protection proceedings to vaccinate against a parent’s will (67). Another is to protect patients in the waiting room from the risks posed by non-vaccinating patients, which is used to justify dismissing non-vaccinating patients from practice (9,26,38, 40,45). Counter-obligations are used to argue against practice dismissal. These include a health professional’s obligation to provide healthcare in the best interest of the child despite the parent’s decisions, and to deal with infectious disease as a part of their role (9,26,45,47, 56,101). Authors also argue that physicians’ obligations *exclude* enforcing parental accountability through dismissal, especially if that means the child is held accountable for the actions of their parents (47), and that continuing to provide care to a non-vaccinating family does not make the physician complicit in their decision (116).

It is sometimes asserted that the state is obliged to discourage non-vaccination on a number of grounds. This includes a fundamental duty of states to protect society [21], a responsibility of states to protect herd immunity as a common good or to reduce social and financial burdens and costs (53,119), and the state’s role to protect the common good in the face of risks to public health and the fallibility of individuals’ risk perception (54). Some of these arguments focus on exemptions from mandatory vaccination policies, proposing that states can not justify such exemptions because the government’s interest in protecting society outweighs the individual’s interest [21] or because vaccination is a social and moral good owed by a society to its children (118).

## Discussion

This review systematically explored and characterised the normative arguments made about parental refusal of routine childhood vaccination. Included publications addressed two types of arguments (i) ‘Refusal’ arguments (whether vaccine refusal is justified) and (ii) ‘Response’ arguments (whether various policy or practice responses to those who reject vaccines are justified). There were more ‘response’ arguments than ‘refusal’ arguments in the literature. On balance, most of the literature on ‘refusal’ arguments contended that it is not justifiable for parents to refuse vaccination for their children. Most of the ‘response’ argument literature argued against the various responses to non-vaccination put forward. However, compared to ‘refusal’ arguments, ‘response’ arguments were more varied and nuanced, and often came with caveats (e.g. exemptions to mandates are permissible *if* the disease burden is low).

The included articles predominantly originated from medical journals: these accounted for most of the papers focused on ‘response’ arguments. This may arise from the broader distribution of academic literature – there are more papers published in medicine than in the other disciplines represented in this review. It may also reflect the needs of readers of medical literature for guidance on how they should respond to non-vaccinating parents, highlighting the importance of making literature addressing the ethical dimensions of vaccine refusal accessible to immunization practitioners. Although there were some interdisciplinary perspectives, the dominance of the medical literature relating to ‘response’ arguments suggests that knowledge in this field may be advanced by incorporating more voices with expertise in ethics, law, and policy. This is especially important for deciding how to implement policy and practice responses to non-vaccination.

‘Refusal’ arguments were more common in the comparatively smaller collection of ethics/philosophy literature identified by this search, which may be, in part, a product of the differences in disciplinary traditions. While ethics/philosophy texts explore counterarguments and reach conclusions that are nuanced, and often with caveats, medical disciplines are primarily guided by practical considerations and a tradition of arguing from evidence rather than from ethical or philosophical principles. This privileging of evidence over principles may make it difficult to explore differing vaccination positions within the medical arena, potentially contributing to the adversarial clinical immunisation encounters described by vaccine-refusing parents and clinicians alike [7, 18, 19]. This pattern needs attention if ethical arguments are to have an impact in practice. As shown, most ethical arguments pay attention to evidence, as most ethical arguments include consequences in some way (see below). Ethical arguments can add nuance to biomedical thinking about consequences (e.g. consequences for individuals vs. the collective) and also about competing values (e.g. balancing consequences against concerns regarding autonomy, consent and liberty). The challenge for ethicists is to provide these arguments in an accessible and compelling form.

In fact, (i) consequences for the child and others, and (ii) respect for autonomy, the doctrine of informed consent and the value of liberty were dominant themes in both ‘refusal’ and ‘response’ arguments. Arguments were guided by common concepts such as the value of herd immunity, the prospect of harm to the child or others in the community and legal perspectives and precedents. The normative significance of parental trust, distrust, and uncertainty was a consideration unique to the ‘refusal’ arguments literature, driven in part by the five parental accounts from the special issue of *Narrative Inquiry in Bioethics* included in our sample. The concepts of inequity, and the duties of governments and practitioners only appeared in ‘response’ arguments. This is unsurprising: it reflects the purpose and perspective of these writers. An analysis of policy options is often required to bring inequity into view, and both clinicians and policymakers have obligations by virtue of their roles that can inform thinking about the right thing to do.

Many of the arguments justifying vaccine refusal aligned with the wider literature on the perspectives of non-vaccinating parents who value the freedom to make health decisions as caregivers, in what they perceive to be the best interest of their children [20, 21]. These decisions are often based on doubts about vaccine safety or efficacy and are commonly initiated by a negative experience [19, 20, 22]. Unsurprisingly, arguments against rejecting childhood vaccines reflected the broader literature on how vaccine-supporting people view non-vaccination— including views that non-vaccinators are misinformed and disrupt social order, and that their actions are not based on reason or shared social values [23]. Common negative descriptors such as “anti-vaxxer” have similar valence in social discourse [24]. Those writing about vaccination should be aware of the potential for stigmatization and “othering” that can result by framing non-vaccination as a failure of parents [25]. When such arguments are used to inform policy and practice responses to non-vaccination, it introduces the potential for negative psychosocial impacts and further alienation of non-vaccinating parents.

Most ‘response’ arguments dealt with the justifiability of mandates and coercive policy. Generally, authors in favour of mandates prioritised the good of society; those against mandates prioritised individual choice. The large number of papers we found on mandates is unsurprising, given that these policies have been contentious. In Australia, federal and most state governments have mandates that require children to be vaccinated to be enrolled in childcare and for their families to be eligible for government financial assistance [26] Key political, academic and industry stakeholders argue that these mandates are designed to increase vaccination rates for the benefit of society [27]. On the other hand, Australian non-vaccinating parents express a belief that their children do not pose a threat to society, that all children should be treated in the same way, and that all parents should be able to make decisions for their children, regardless of vaccination status [28]. These perceptions of policy makers and non-vaccinating parents broadly represent the opposing arguments about mandates presented in this review. Facilitating a middle-ground approach to policy implementation may require closer attention to the values underlying these opposing views, and using a procedurally just approach to weigh them against one another.

In the context of an increasing number of systematic reviews in the field of bioethics, there has been recent criticism emerging about the use of these methods in bioethics. For example, Birchley and Ives (2022) argue that such methods are designed and therefore better suited to aggregation of quantitative data and not the complex and subjective nature of bioethical concepts and the theory-generating and interpretive approaches they require [29]. We argue that our application of the framework systematic review method - one of many well-established methods for systematic review and synthesis of qualitative and conceptual data - is appropriate for this research question and the application of our findings. Vaccine policy and practice requires a synthesis of what is known on relevant issues, and a systematic approach such as that used here provides a useful summary of the breadth of relevant ethical issues in a format that is accessible to policymakers. Our review has some limitations. Our aim was to map the range of normative arguments about vaccination refusal and policy. We did not have scope to present a novel ethical argument in response to our findings; this is an aim for future empirical and theoretical research. Most of the included literature focuses on high-income settings, predominantly the United States and the United Kingdom. In low-income settings, health services are often harder to access and levels of and reasons for vaccine rejection also differ in these settings. For example, political and cultural factors have been implicated in polio vaccine rejection in Nigeria [30], while low literacy, unemployment, and owning a mobile phone have been associated with polio vaccine refusal in Pakistan [31]. Our sampling period included a special issue of *Narrative Enquiry in Bioethics* which published narratives written by parents to communicate their normative positions on vaccination. These were mostly written by non-vaccinating parents and made up over one third of all arguments in the literature that support refusal. This is a strength in that it expanded the range of views represented in the review. However, it is also a limitation in that if this special issue had not been published within our sampling period, the range of arguments would have been more strongly skewed against vaccine refusal. These papers artificially increased the proportion of arguments in the scholarly domain that argue for vaccine refusal. It is a strength of our methodology that we were able to identify the unique perspective from which they were written and position them separately in our literature synthesis so that our representation of the literature distribution is not artificially skewed.

## Conclusion

This review highlights an opportunity for interdisciplinary collaboration to widen the scope and reach of normative arguments about non-vaccination. Such collaboration can facilitate a broader understanding of and engagement with the ethical issues that may be relevant for practitioners, policymakers, and researchers in deciding how to respond to non-vaccinating parents. Arguments about the justifiability of non-vaccination and what should be done about it have the potential to positively influence routine childhood vaccination rates but can also alienate non-vaccinating families if not deployed with their perspectives in mind. There is an avenue for future work to further understand the influence of cultural context on normative arguments, especially within low- and middle-income settings. Moreover, there is an opportunity to further explore the influence and translation of scholarly ethical arguments into policy and practice responses to childhood non-vaccination.

## Data Availability

The datasets generated and/or analysed during the current review are not publicly available, however the search terms used to generate the dataset are included in this published article.
